# The Global Antifungal Susceptibility Epidemiology of *Trichophyton indotineae* Indicating Voriconazole as an Alternative Therapy for Recalcitrant Dermatophytosis

**DOI:** 10.1111/myc.70195

**Published:** 2026-06-05

**Authors:** Ge Song, Wenting Xie, Xiaodong She, Weida Liu, Xiaofang Li, Guanzhao Liang

**Affiliations:** ^1^ Department of Dermatology, Beijing Shijitan Hospital Capital Medical University Beijing China; ^2^ Department of Medical Mycology, Hospital for Skin Diseases, Institute of Dermatology Chinese Academy of Medical Sciences and Peking Union Medical College Nanjing China; ^3^ Jiangsu Provincial Key Laboratory of Dermatology Nanjing China; ^4^ CAMS Collection Centre of Pathogen Microorganisms‐D (CAMS‐CCPM‐D) Nanjing China

**Keywords:** antifungal drug resistance, CLSI, EUCAST, *Trichophyton indotineae*, voriconazole

## Abstract

**Background:**

The emergence of *Trichophyton indotineae* as a multidrug‐resistant dermatophyte has challenged conventional therapeutic approaches for dermatophytosis, leading to a significant therapeutic dilemma. The absence of formal epidemiological cutoff values (ECVs) under the Clinical and Laboratory Standards Institute (CLSI) framework has led to inconsistent interpretation of antifungal susceptibility testing (AFST) data, highlighting the urgent need for standardised susceptibility interpretation criteria and effective therapeutic strategies.

**Objectives:**

This study aimed to (1) establish CLSI‐based estimated tentative ECVs (ET‐ECVs) to facilitate reliable minimum inhibitory concentration (MIC) interpretation; (2) analyse resistance rates of *T. indotineae* to terbinafine, itraconazole and voriconazole using a large‐scale global dataset and (3) explore and identify effective therapeutic options following first‐line treatment failure.

**Methods:**

This systematic review compiled global susceptibility data for *T. indotineae* through comprehensive literature searches, including only studies that performed CLSI‐ or European Committee on Antimicrobial Susceptibility Testing (EUCAST)‐standardised AFST. Furthermore, the clinical efficacy of voriconazole was specifically evaluated by summarising and analysing reported treatment cases.

**Results:**

A total of 67 studies from 14 countries, comprising 981 isolates, were included. The proposed CLSI ET‐ECVs for terbinafine, itraconazole and voriconazole were 0.125, 0.25 and 1 mg/L, respectively, showing full alignment with EUCAST tentative epidemiological cutoff values (TECOFFs). Integrated resistance rates were 77.6% for terbinafine, 17.0% for itraconazole and 1.4% for voriconazole. Cross‐resistance analysis revealed that terbinafine resistance predominated, with 14.3% of isolates co‐resistant to itraconazole. In vitro susceptibility data demonstrated high activity of voriconazole and clinical case summaries further confirmed its favourable therapeutic efficacy, with complete remission reported in all evaluated cases.

**Conclusion:**

This study defined the CLSI ET‐ECVs for interpreting *T. indotineae* MICs, highlighted the resistance burden to first‐line agents and provided in vitro evidence supporting further evaluation of voriconazole as a potential effective alternative therapy. These findings may provide critical warnings regarding the high‐resistance landscape and help guide antifungal susceptibility interpretation for this emerging multidrug‐resistant pathogen.

## 1 Introduction

Dermatophytosis has long been considered a superficial fungal infection that responds well to standard therapy. Over the past decade, however, this paradigm has shifted with the emergence of *Trichophyton indotineae* (previously *T. mentagrophytes* ITS genotype VIII), a rapidly spreading, terbinafine‐resistant dermatophyte now recognised as a global public health concern [1, 2]. First detected predominantly in South Asia—particularly India [3]—the pathogen has since been reported in more than 40 countries across Asia, Europe, the Americas, Africa and Oceania [4, 5, 6]. In addition to travel‐associated importation, autochthonous transmission, household clustering and sexually associated spread have been documented, highlighting its substantial transmissibility [7]. Clinically, *T. indotineae* causes chronic, recalcitrant and often extensive tinea corporis, cruris and faciei, typically accompanied by severe pruritus and significant impairment in quality of life [8]. Its rapid geographic expansion and distinctive clinical phenotype have transformed what was once a regional problem into a global challenge.

Equally concerning is its broad resistance to commonly used antifungals. Terbinafine has historically been the first‐line treatment for *Trichophyton* infections because of its fungicidal activity, favourable safety profile and excellent tissue penetration. However, numerous investigations now report markedly elevated terbinafine minimum inhibitory concentration (MIC) values and frequent clinical failure in *T. indotineae* infections [9, 10]. We have finished one large genomic epidemiology study that identified terbinafine resistance in approximately 69% of isolates [11]. As terbinafine efficacy diminishes, itraconazole and other azoles have become commonly used alternatives; yet real‐world experience suggests that high doses (0.2 g twice daily) and/or prolonged treatment courses (> 2 weeks) are often required, raising concerns regarding toxicity, drug–drug interactions and cost [12]. Reduced susceptibility to fluconazole and itraconazole—reported at roughly 68% and 28%, respectively—further limits therapeutic options [11]. Notably, our recent isolates from China include multiple multidrug‐resistant strains showing resistance to terbinafine, fluconazole, griseofulvin and, in some cases, itraconazole or posaconazole, while remaining consistently susceptible to voriconazole, underscoring its potential as an alternative agent, while it lacks sufficient globally supported evidence [13]. Understanding evolving susceptibility patterns and optimising antifungal selection have therefore become priorities.

Despite rising global resistance, standardised interpretive criteria for antifungal susceptibility testing (AFST) remain limited [14]. Although broth microdilution methods—primarily those outlined in Clinical and Laboratory Standards Institute (CLSI) M38 [15], along with European Committee on Antimicrobial Susceptibility Testing (EUCAST) E.Def 11.0 [16]—are widely used to determine MICs, no validated CLSI clinical breakpoints or epidemiological cutoff values (ECVs) are currently available for *T. indotineae* [17]. EUCAST has introduced tentative epidemiological cutoff values (TECOFFs) for several antifungal agents, providing an important initial framework for distinguishing wild‐type from non–wild‐type isolates; however, their provisional status and limited clinical validation mean that further refinement is needed before they can fully inform routine patient management [18]. Consequently, interpreting AFST results remains challenging, creating a critical gap between laboratory measurements and therapeutic decision‐making.

To address these needs, we systematically compiled global MIC data for *T. indotineae* from studies using CLSI‐ or EUCAST‐compliant microdilution methods and applied a nano‐exclusion analytical approach to derive estimated tentative CLSI ECVs. Particular emphasis was placed on voriconazole, an azole with promising therapeutic potential but limited prior MIC characterisation. Collectively, this work could summarise the resistance profile, refine the interpretive criteria and identified an alternative treatment option for recalcitrant dermatophytosis.

## 2 Materials and Methods

To comprehensively assess the global drug resistance profile of *T. indotineae* and evaluate the efficacy of voriconazole in treating drug‐resistant strain infections, we conducted a systematic review of existing literature. A comprehensive literature search was performed using PubMed, Scopus, Embase and Web of Science with the following key terms: (‘*T. indotineae*’ OR ‘*T. interdigitale*’ OR ‘*T. mentagrophytes*’) AND (‘drug resistance’ OR ‘terbinafine resistance’ OR ‘azole resistance’ OR ‘voriconazole’).

The search was restricted to English‐language publications meeting the following inclusion criteria: (i) *T. indotineae* (*T. mentagrophytes* ITS genotype VIII) infection confirmed by fungal culture and molecular identification; (ii) AFST performed in accordance with CLSI M38 [15] or EUCAST [16] protocol; (iii) availability of complete AFST results with explicit MIC values and (iv) for studies reporting voriconazole treatment, provision of detailed therapeutic information. All included articles underwent rigorous methodological validation, with particular attention to technical details of antifungal susceptibility testing. Studies were excluded based on the following criteria to ensure data comparability: (i) AFST procedures deviated from CLSI M38 or EUCAST standards, particularly regarding inoculum preparation (e.g., conidial suspension density), MIC endpoint determination or incubation temperature (defined as > 5°C deviation from guideline specifications) and (ii) the dataset overlapped with previously published studies or contained duplicate patient cohorts.

For all eligible studies, a predesigned data extraction sheet was used to systematically record the following information: author details, country of origin, publication year, causative pathogen, infection site, genetic mutations and antifungal susceptibility profiles. Due to heterogeneity in data reporting across studies, relevant and comparable data were selectively extracted for analysis. For MIC values that were not precisely determined (e.g., reported as inequalities), data were adjusted as follows: values reported as ‘≥’ (greater than or equal to) or ‘≤’ (less than or equal to) were assigned the specific value itself. Values reported as ‘>’ (greater than) or ‘<’ (less than) were assigned the value of the next highest or lowest two‐fold dilution concentration, respectively for numerical analysis. The entire search and selection process was independently conducted by two investigators, with any discrepancies resolved through discussion with a third reviewer.

Statistical analysis and graphical presentation of the data were performed using GraphPad Prism software (version 10.0, San Diego, CA, USA). Descriptive statistics were used to summarise the MIC distributions, and the estimated tentative ECV was applied to derive putative resistance rates. In this study, “resistance rate” refers to the proportion of isolates exceeding the estimated tentative ECV, rather than clinical resistance defined by established breakpoints. Venn diagrams were constructed to visually represent the overlap and unique occurrences of resistance to terbinafine, itraconazole and voriconazole among the clinical isolates. The proportional areas of the circles in the Venn diagrams were adjusted to reflect the respective sample sizes for each drug combination, providing an accurate visualisation of the cross‐resistance patterns.

## 3 Results

This systematic review incorporated a total of 67 studies, involving 14 countries across Asia, Europe, North America and South America, with MIC values available for 981 fungal isolates.

### 3.1 Resistance Rates of *T. indotineae* to Terbinafine, Itraconazole and Voriconazole Based on EUCAST

**FIGURE 1:**
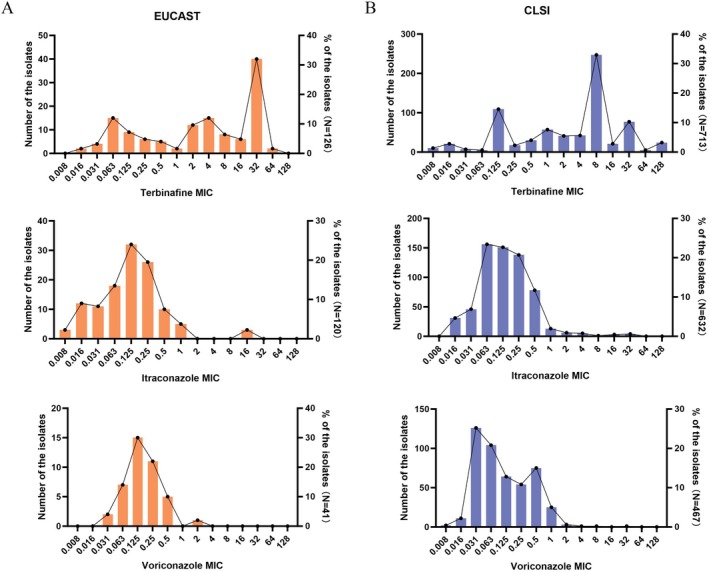
Comparative minimum inhibitory concentration (MIC) distributions of terbinafine, itraconazole and voriconazole against *Trichophyton indotineae* isolates under European Committee on Antimicrobial Susceptibility Testing (EUCAST) and Clinical and Laboratory Standards Institute (CLSI) standardised methods. MIC distributions for terbinafine, itraconazole and voriconazole are shown, with data generated according to EUCAST (A) and CLSI (B) methodologies. In each panel, bar graphs indicate the number of isolates at each MIC, and overlaid line graphs represent the cumulative percentage. MICs are expressed in mg/L.

The MIC values of terbinafine, itraconazole and voriconazole were determined for 126, 120 and 41 isolates of *T. indotineae*, respectively, using the EUCAST method (Figure 1A). Based on the tentative epidemiological cut‐off values (TECOFFs) established for 0.125 mg/L for terbinafine, 0.25 mg/L for itraconazole and 1 mg/L for voriconazole, the resistance rates were calculated to be 76.2%, 15.0% and 2.4%, respectively. A striking 31.8% of terbinafine‐resistant isolates demonstrated a high MIC of 32 mg/L, implying that dose escalation is an unlikely solution, which aligns with clinical reports of high‐dose treatment failures. Moreover, the MICs of susceptible isolates clustered between 0.063 and 0.125 mg/L, immediately adjacent to the ECOFF, indicating they are on the precipice of resistance and may develop resistance under drug selection pressure. In contrast, itraconazole and voriconazole displayed continuous, unimodal MIC distributions, with 65.0% and 80.5% of values, respectively, falling between 0.063 and 0.25 mg/L, suggesting that the observed resistance is likely mediated by polygenic or adaptive mechanisms.

### 3.2 Determined CLSI ET‐ECV and Resistance Rates of *T. indotineae* to Terbinafine, Itraconazole and Voriconazole

The MIC values of terbinafine, itraconazole and voriconazole were determined for 713, 632 and 467 isolates of *T. indotineae*, respectively, using the CLSI method (Figure 1B). EUCAST and CLSI represent the internationally recognised reference standards for antifungal susceptibility testing, with a well‐documented high degree of concordance. Leveraging this established consistency and addressing the current absence of CLSI‐defined ECV, we employed a derivation method founded on the following principle: the resistance rates calculated using EUCAST criteria may closely approximate those derived from CLSI criteria for the same dataset. Based on this approach, we defined the estimated tentative ECV (ET‐ECV) for CLSI as 0.125 mg/L for terbinafine, 0.25 mg/L for itraconazole and 1 mg/L for voriconazole. Interestingly, the values we determined were fully consistent with established EUCAST TECOFF. Hence, in the CLSI dataset, the respective resistance rates for terbinafine, itraconazole and voriconazole were 78.7%, 17.4% and 1.3%. The combined analysis of EUCAST and CLSI data yielded integrated resistance rates of 77.6% for terbinafine, 17.0% for itraconazole and 1.4% for voriconazole.

Analysis of the MIC distribution showed that the terbinafine‐resistant isolates clustered at MICs of 8 mg/L (34.6%) and 32 mg/L (10.8%), while the susceptible wild‐type was concentrated at an MIC of 0.125 mg/L (15.3%), indicating that increasing the dose or duration of terbinafine is unlikely to achieve cure—a notion consistent with clinical reports of treatment failure even under high‐dose regimens. For itraconazole, the MICs of non‐resistant isolates clustered between 0.063 and 0.25 mg/L and resistant isolates primarily exhibited an MIC of 0.5 mg/L, a value immediately adjacent to the ET‐ECV. This proximity suggests that resistance may be overcome by optimising the dosing regimen, providing a rationale for the clinically observed efficacy of dose escalation or extended treatment. Voriconazole MICs were similarly clustered within the wild‐type range (0.063–0.25 mg/L), indicating a predominantly susceptible population.

### 3.3 Co‐Resistance Profile of Terbinafine and Itraconazole

**FIGURE 2:**
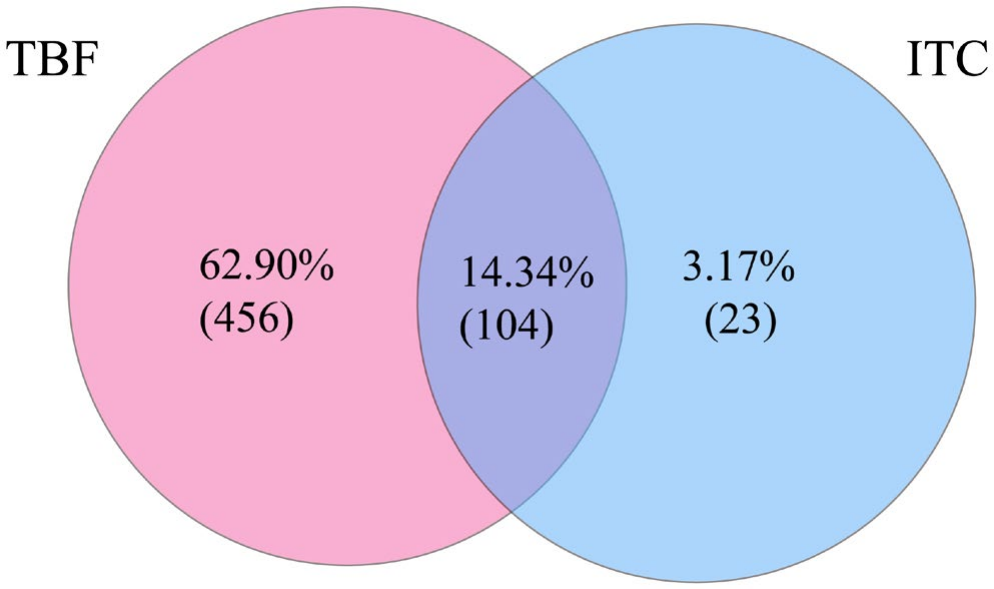
Venn diagram illustrating the overlap of resistance to terbinafine (TBF) and itraconazole (ITC).

A total of 725 isolates with available MIC values for both terbinafine and itraconazole were analysed, including those tested by CLSI or EUCAST methods (Figure 2). The vast majority (63.0%, 456/725) exhibited resistance exclusively to terbinafine, indicating a high prevalence of solitary terbinafine resistance. A proportion of isolates (14.3%, 104/725) demonstrated co‐resistance to both agents. In contrast, resistance to itraconazole alone was uncommon (3.2%, 23/725). This pattern establishes terbinafine resistance as the dominant phenotype, with a minority of cases exhibiting co‐resistance to itraconazole. This observation points to an evolutionary trajectory from terbinafine‐specific to multi‐drug resistance in *T. indotineae* under sustained drug pressure.

### 3.4 Multidrug Resistance Profile of Terbinafine, Itraconazole and Voriconazole

**FIGURE 3:**
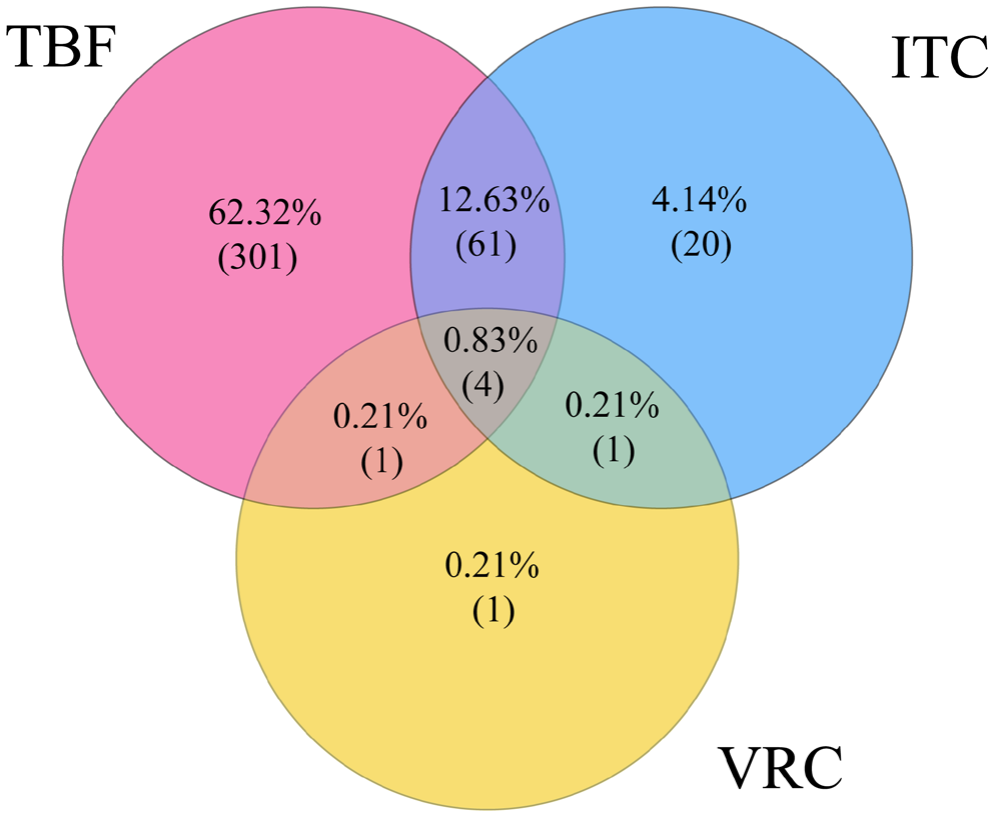
Venn diagram illustrating the overlap of resistance to terbinafine (TBF), itraconazole (ITC) and voriconazole (VRC).

A total of 482 isolates with simultaneously determined MIC values for terbinafine, itraconazole and voriconazole were analysed, incorporating data from both CLSI and EUCAST methodologies (Figure 3). Terbinafine resistance was predominant, with the majority of isolates (62.5%, 301/482) exhibiting resistance to terbinafine alone. Co‐resistance to both terbinafine and itraconazole was observed in 12.7% (61/482) of isolates. Pan‐resistance to all three antifungals (terbinafine, itraconazole and voriconazole) was rare, detected in only four isolates (0.8%). Resistance to both terbinafine and voriconazole, as well as to itraconazole and voriconazole, was observed in merely 1 isolate each. The solitary resistance rates for itraconazole and voriconazole were 4.1% and 0.2%, respectively. These data underscore the high prevalence of terbinafine resistance and a growing trend of co‐resistance to terbinafine and itraconazole. Therefore, the in vitro susceptibility data collectively indicate that voriconazole retains potent activity against the vast majority of *T. indotineae*, including those resistant to terbinafine and itraconazole.

### 3.5 Voriconazole Can Be Alternative Treatment for Drug‐Resistant *T. indotineae* Infections

Based on the aforementioned findings demonstrating a low resistance rate of voriconazole, it is suggested that voriconazole could be a promising new therapeutic alternative for refractory dermatophytosis. We have summarised successfully treated cases of refractory *T. indotineae* infections using voriconazole (Table 1). Although these are individual case reports, a consistent pattern emerges: prior to initiating voriconazole, all patients had received multiple conventional antifungal agents (e.g., terbinafine, itraconazole, griseofulvin) resulting only in treatment failure or partial response, underscoring the refractory nature of their conditions. Following the switch to voriconazole, all cases achieved complete remission. The successful application of voriconazole via both oral and topical routes demonstrates its dosing flexibility, allowing for individualised treatment strategies tailored to the infection site and severity. This consistent successful outcome robustly confirms the efficacy of voriconazole for refractory dermatophytosis in clinical practice.

**TABLE 1:** Summary of clinical cases with *Trichophyton indotineae* infections treated with voriconazole: Patient characteristics, treatment history and outcomes.

No.	Country	Publication time	Cases number	Sex/age	Gene mutations	Site of infection	Duration	Primary treatment	Outcome	Final treatment	Clinical response	AFST method
1	Israel [19]	2024	2	26/M	Phe397Leu	Tinea cruris	15 months	ITC (200 mg/day, 2 months)	NR	VRC (200 mg/day), 3 months	CR	NA
				28/M	Phe397Leu	Tinea corporis, Tinea cruris	15 months	ITC (200 mg/day, 2 months)	PR	VRC (400 mg/day), 2 months	CR	NA
2	Kuwait [20]	2023	1	33/F	Phe397Leu	Tinea corporis, Tinea cruris, Tinea faciei	NA	TBF (250 mg/day, 6 weeks); GRF (1 g/day, 6 weeks); ITC (400 mg/day, 4 weeks)	NR	VRC (400 mg bid for 2 days), VRC (200 mg bid for 3 months)	CR	EUCAST: TBF = 4, ITC = 0.016, VRC = 0.125, POS = 0.016
3	India [21]	2022	1	24/M	NA	Tinea corporis, Tinea cruris, Tinea faciei	4 years	FCZ (150–300 mg/week), ITC (100‐200 mg/day), TBF (500 mg/day), GRF (500 mg/day) for multiple courses; KCZ (200 mg, q12h); TBF (250 mg q12h, 10 weeks); FCZ (100 mg/day, 11 weeks)	NR; PR; NR; NR	VRC (200 mg/day), 2 weeks	CR	CLSI: TBF = 32, ITC = 0.125, FCZ = 4, POS < 0.015
4	France [22]	2022	1	28/M	Leu393Ser	Tinea corporis, Tinea cruris	NA	TBF (250 mg/day, 30 weeks), GRF (500 mg bid, 9 weeks)	PR	topical 1% VRC cream, 6 months; topical 1% VRC cream, 2 months	CR; CR	NA
5	Iran [7]	2021	1	31/F	Phe397Leu	Tinea corporis, Tinea cruris, Tinea faciei	More than 1 year	FCZ (150 mg/day); TBF (250 mg/day, 1 month); ITC (200 mg bid for 1 week each month)	NR; NR; PR	VRC (200 mg/day), 4 weeks	CR	CLSI: TBF ≥ 8, ITC ≥ 4, FCZ ≥ 16, VRC = 0.25–0.5, POS = 0.313–0.006

Abbreviations: AST, antifungal susceptibility testing; bid, twice daily; CLSI, Clinical and Laboratory Standards Institute; CR, complete response; EUCAST, European Committee on Antimicrobial Susceptibility Testing; F, female; FCZ, fluconazole; GRF, griseofulvin; ITC, itraconazole; KCZ, ketoconazole; M, male; MIC, minimum inhibitory concentration; NA, not available; NR, not reported; POS, posaconazole; PR, partial response; TBF, terbinafine; VRC, voriconazole.

## 4 Discussion

The rising prevalence of drug‐resistant dermatophytes, particularly *T. indotineae*, presents a growing public health challenge, necessitating a deeper understanding of its resistance landscape [23, 24]. This study synthesises global susceptibility data to characterise a clear resistance pattern: *T. indotineae* demonstrates widespread, high‐level resistance to terbinafine, coupled with a proportion of co‐resistance to itraconazole, alongside emerging multidrug resistance. In this context of increasingly constrained therapeutic options, voriconazole presents as a potent and effective alternative, supported by its consistently low resistance rates and documented clinical efficacy, positioning it as a leading candidate for managing these recalcitrant infections.

This study evaluated the resistance profiles of *T. indotineae* to three commonly used antifungal agents. In contrast to earlier reports, we applied strict inclusion criteria, incorporating only susceptibility tests that fully complied with CLSI or EUCAST reference methods [25, 26]. This approach minimised biases associated with methodological variability and provided a more reliable assessment of resistance trends. The analysed dataset revealed a high prevalence of resistance to terbinafine, with 77.59% of isolates classified as resistant. A substantial proportion of these exhibited elevated MIC values (8–32 mg/L), indicating high resistance. Furthermore, most resistant strains harbour missense mutations in the squalene epoxidase (SQLE) gene—corroborating a well‐defined molecular basis for resistance, implying that dose escalation is unlikely to overcome resistance [27, 28].

Given the limited efficacy of terbinafine, itraconazole has become a first‐line alternative [29]. However, resistance to itraconazole is increasingly reported, and this study showed the resistance rate reached 17.0%. Importantly, co‐resistance between terbinafine and itraconazole was observed in 14.3% of isolates. Azole resistance involves multiple mechanisms, including increased *cyp51b* copy number, overexpression of efflux pumps, and so on [24, 30, 31]. Prolonged use of itraconazole exerts antifungal selective pressure that may promote tolerance and eventual resistance, raising the risk of therapeutic failure [19]. In cases where both first‐line agents are ineffective, alternative treatment strategies are urgently needed.

Voriconazole demonstrates significant therapeutic value in managing *T. indotineae*‐associated resistant dermatophytosis. In vitro susceptibility testing showed a notably low resistance rate to voriconazole (1.3%–2.4%), significantly lower than rates for terbinafine and itraconazole. Most isolates, including those resistant to first‐line agents, exhibited low MIC values against voriconazole, demonstrating its potent in vitro activity. Clinically, voriconazole has proven highly effective as salvage therapy by multiple case reports and studies [7, 19, 20, 21, 22]. Shahzad et al. [32] reported in a study of 200 patients that voriconazole showed an overall effectiveness of 82% for tinea corporis/cruris, with 90%, 71% and 58% efficacy in new, recurrent and drug‐resistant cases, respectively. Khattab et al. [33] in a study on recalcitrant dermatophytosis indicated that after a 6‐week treatment, voriconazole monotherapy (800 mg loading dose followed by 400 mg daily) yielded significantly higher clinical cure (83.3%) and mycological cure (86.7%) rates than itraconazole monotherapy (200 mg/day) or its combination with isotretinoin (200 mg/day + 20 mg/day), with lower relapse. Similarly, Chandrashekar and Poojitha [34] documented that a two‐week course of voriconazole (800 mg loading dose on Day 1, then 400 mg daily) resulted in complete clearance rates of 90% and 75% at Weeks 2 and 6 post‐treatment, with a 5% relapse rate. Ali et al. [35] also demonstrated a significantly higher complete response rate for voriconazole (200 mg twice daily) than itraconazole (100 mg twice daily) following a 2–4 week course (84.2% vs. 15.7%). Voriconazole holds critical candidate in the management of recalcitrant dermatophytosis by providing a potent and reliable solution. Based on pharmacokinetic and pharmacodynamic principles, it is recommended to prioritise an initial oral loading dose on the first day, followed by a maintenance dose, to ensure rapid and effective antifungal exposure. To optimise treatment outcomes and minimise risks, therapeutic drug monitoring (TDM)‐guided dosing represents a feasible strategy. Evidence indicates that trough concentrations > 0.5 mg/L ensure efficacy, while levels > 3.0–4.0 mg/L increase toxicity risks, suggesting TDM has practical value in supporting future medication safety [36]. It is important to note that voriconazole is associated with common adverse effects such as hepatotoxicity, visual disturbances and photosensitivity, as well as significant drug–drug interactions, mandating regular monitoring during therapy [37].

Currently, AFST for *T. indotineae* is predominantly performed using the CLSI methodology. However, the lack of official ECV within this framework has resulted in inconsistent resistance interpretation across studies, impairing both cross‐study comparability and the standardisation of clinical guidance [14, 17]. To bridge this gap, the present study draws upon the established high concordance between EUCAST and CLSI systems to propose a set of exploratory ET‐ECV derived from CLSI data. The proposed CLSI ET‐ECV align exactly with established EUCAST TECOFFs [18], and the resistance rates calculated using these values showed strong agreement with those generated by EUCAST criteria. This consistency underscored the reliability of the proposed ET‐ECV and provided a scientifically sound basis for MIC interpretation in laboratories using CLSI methods, thereby addressing a significant limitation in current practice. We fully acknowledge that this method, though innovative and promising, does not substitute for formally validated breakpoints. While it offers a rational interim strategy in the absence of standardised criteria, further validation is needed to confirm its precision. Ultimately, resolving this issue will require future prospective, multicentre studies with rigorous quality control to establish evidence‐based ECV and clinical breakpoints.

This study also has limitations. The analysis is based on aggregated data from multiple studies, which introduces inherent heterogeneity in isolate collection, laboratory methods and data reporting. Furthermore, the clinical evidence for voriconazole is currently based on case reports and small series, which are subject to publication bias. Nevertheless, the consistency of the in vitro data with the successful clinical outcomes provides a compelling argument for its use.

In summary, this study integrates globally sourced MIC data to address three core objectives: (1) establishing CLSI‐based ET‐ECV to improve the interpretation of AFST and enable consistent definition of resistance phenotypes; (2) comprehensively characterising the antifungal resistance features of *T. indotineae*, including resistance rates to terbinafine, itraconazole and voriconazole, along with cross‐resistance trends and (3) evaluating the evidence for voriconazole as a promising therapeutic alternative.

Through this work, the study aims to inform clinical decision‐making, advance drug resistance surveillance and promote the establishment of standardised interpretive criteria, thereby clarifying the essential role of voriconazole in the management of refractory dermatophytosis.

## Author Contributions

**Ge Song:** methodology, formal analysis, writing – original draft. **Wenting Xie:** validation, software, data curation. **Xiaodong She:** investigation, visualization. **Weida Liu:** resources, supervision. **Xiaofang Li:** writing – review and editing, funding acquisition. **Guanzhao Liang:** conceptualization, writing – review and editing, project administration.

## Funding

This work was supported by CAMS Innovation Fund for Medical Sciences (CIFMS, 2025‐I2M‐C&T‐B‐080 and 2021‐I2M‐1‐039); National Key Research and Development Program of China (2022YFC2504804); Natural Science Foundation Program of Beijing, China (7244363); and Jiangsu Provincial Medical Key Laboratory, Jiangsu Province Capability Improvement Project through Science, Technology and Education (ZDXYS202204).

## Conflicts of Interest

The authors declare no conflicts of interest.

## Data Availability

All data generated or analysed during this study are included in this published article. The datasets supporting the findings are available within the paper and its Table 1 and Figures 1, 2, 3.
